# Supplementary material for: Clinical Outcomes Associated with Parenteral Nutrition Caloric Provision in Geriatric Patients with Infectious Colitis
Source: Nutrients. 2025 Nov 26;17(23):3707. doi: 10.3390/nu17233707 (PMC12694275; doi:10.3390/nu17233707)
Supplement: Supplementary file 1 [file nutrients-17-03707-s001.zip › nutrients-3982211-supplementary.pdf]

Supplementary Table S1. Risk factors for prolonged hospitalization in non-ICU patients (n=239)

Characteristics			Univariate Analysis			Multivariable Analysis		
	Total (n=239)	> 4 days n (%)	OR	95% CI	*p*-value	OR	95% CI	*p*-value
Age > 75 years	104	37 (35.6)	1	0.59 – 1.71	0.997			
Sex (Female)	163	61 (37.4)	1.3	0.73 – 2.31	0.38			
Body mass index ≤ 25 kg/m²	161	66 (41.0)	2.16	1.18 – 3.95	0.013	1.95	1.04 – 3.67	0.038
Long-term care hospital	8	7 (87.5)	13.73	1.66 – 113.59	0.015	10.77	1.24 – 93.26	0.031
Intensive care unit	0	0 (0.0)	–	–	–			
Diabetes	83	32 (38.6)	1.22	0.70 – 2.12	0.482			
Liver disease	13	5 (38.5)	1.14	0.36 – 3.60	0.823			
Malignancy	0	0 (0.0)	–	–	–			
Chronic kidney disease	0	0 (0.0)	–	–	–			
Congestive heart failure	30	9 (30.0)	0.75	0.33 – 1.72	0.497			
PAOD	4	0 (0.0)	0	0.00 – Inf	0.999			
COPD	6	4 (66.7)	3.75	0.67 – 20.93	0.131			
Cerebral vascular attack	32	14 (43.8)	1.49	0.70 – 3.17	0.301			
Hemiplegia	0	0 (0.0)	–	–	–			
Rheumatic disease	7	3 (42.9)	1.37	0.30 – 6.28	0.684			
Dementia	13	7 (53.8)	2.21	0.72 – 6.81	0.166			
Peptic ulcer	5	0 (0.0)	–	–	–			
Charlson comorbidity index > 3	16	8 (50.0)	1.9	0.69 – 5.25	0.218			
Shock	12	3 (25.0)	0.59	0.16 – 2.24	0.438			
WBC > 10,000 or ≤ 4,000 /mm³	131	53 (40.5)	1.61	0.94 – 2.77	0.083			
Hemoglobin ≤ 12 g/dL	83	29 (34.9)	0.96	0.55 – 1.67	0.883			
Platelet count ≤ 150 x 10³/mm³	44	16 (36.4)	1.04	0.53 – 2.06	0.902			

ESR > 22 mm/h	139	58 (41.7)	1.94	1.11 – 3.37	0.02	1.94	1.08 – 3.47	0.026
Lymphocyte count ≤ 1,500 /mm³	172	68 (39.5)	1.92	1.02 – 3.61	0.042	1.87	0.96 – 3.63	0.064
Albumin ≤ 3.3 g/dL	48	24 (50.0)	2.13	1.12 – 4.05	0.021			
Calories per day ≤ 1000 kcal/day	155	62 (40.0)	1.77	0.99 – 3.15	0.053	1.82	0.99 – 3.33	0.054

PAOD: peripheral arterial occlusive disease, COPD: chronic obstructive pulmonary disease, ESR: erythrocyte sedimentation rate
